# Integrated Information as a Measure of Cognitive Processes in Coupled Genetic Repressilators

**DOI:** 10.3390/e21040382

**Published:** 2019-04-10

**Authors:** Luis Abrego, Alexey Zaikin

**Affiliations:** 1 Department of Mathematics, University College London, London WC1E 6BT, UK; 2 The Alan Turing Institute, London NW1 2DB, UK; 3 Institute for Women’s Health, University College London, London WC1E 6BT, UK; 4 Department of Applied Mathematics and Laboratory of Systems Biology of Aging, Lobachevsky State University of Nizhniy Novgorod, 603022 Nizhniy Novgorod, Russia; 5 Department of Pediatrics, Faculty of Pediatrics, Sechenov University, 119146 Moscow, Russia

**Keywords:** integrated information, complex dynamics, synthetic biology, genetic regulatory networks, repressilator, cell differentiation, synchronization, clustering

## Abstract

Intercellular communication and its coordination allow cells to exhibit multistability as a form of adaptation. This conveys information processing from intracellular signaling networks enabling self-organization between other cells, typically involving mechanisms associated with cognitive systems. How information is integrated in a functional manner and its relationship with the different cell fates is still unclear. In parallel, drawn originally from studies on neuroscience, integrated information proposes an approach to quantify the balance between integration and differentiation in the causal dynamics among the elements in any interacting system. In this work, such an approach is considered to study the dynamical complexity in a genetic network of repressilators coupled by quorum sensing. Several attractors under different conditions are identified and related to proposed measures of integrated information to have an insight into the collective interaction and functional differentiation in cells. This research particularly accounts for the open question about the coding and information transmission in genetic systems.

## 1. Introduction

Information-processing in living systems spans different degrees of biological complexity. From neural networks, where we find integration of information from different segregated modules in the nervous system, to the gene regulation and cell differentiation, whose structural mechanisms allow integration of signal transduction processes for survival and adaptation. Growing evidence shows that this degree of integration of stimuli is a required step to exhibit consciousness in the human brain where the information one receives is segregated and stored and later integrated and displayed.

These underlying mechanisms of information-processing in complex vertebrates can also be observed in simpler organisms such as unicellular organisms. As reviewed by [1], bacteria hold the ability for receiving, processing, and encoding information from the environment. This is done through inputs from different biochemical entities sensed from several signaling pathways. Later, this information is encoded for decision-making. This process can involve causal dependencies such as allocating previous information for calibration of responses or behavior, and even anticipation of stimuli. Thus, it is not surprise that several researchers have stressed the role of unicellular organisms as part of an evolutionary process for the development of the human brain [2,3,4]. Multiple microbiologists suggest that bacteria exhibit cognitive capacities that draw parallels in functionality as well as in the molecular mechanisms, ecology and evolution [1].

In cells, there is a functional integration of information channels coming from a vast repertoire of signaling pathways in which memory, learning, and decision-making take place under a changing environment. At the intracellular level, cells process information through gene regulatory networks, in which each gene influences the behavior of the other. The mechanisms found here serve to transduce the information sensed from the surroundings and the individual demands, including population density, individual cellular stress, stress induced from neighboring cells and the memory of previous individual and multicellular states [5,6,7]. At multicellular level, there is a recursive communication with the surrounding through signaling architectures, which include sensory mechanisms such as cell population density to brain-like electrical signaling. Such an interplay among the different levels of complexity allows processes such as cell differentiation, metabolism and DNA repair [8].

Still, an agreed definition of cognition and its boundaries across living systems remains elusive, and typically the term is weighted against the human case. To date, some authors describe cognition as a set of mechanisms such as information acquisition and coding from the environment [9] that allow biological entities act in a specific way to satisfy a viability constraint [10], or as a complex of sensory and information-processing derived by the structural and causal bidirectional interaction between organism and environment [1,5,11]. These interactions allow coding and processing of information to satisfy the requirements in living systems and provide the ability of acquiring, retaining, and using information from the environment to sensing, learning, and decision-making for adaptation of behavior and physiology for survival and growth. Despite these insights, the biological mechanisms at the cellular level remain unclear. Further investigation is needed to have a better understanding of its capabilities and identify its link with higher organisms.

The question that concerns this discussion is to what extent does the whole regulatory system in genetics have a balance between integration (coherence among the components of the system) and segregation (components dynamically independent) or to what extent is the whole system generating more information than the sum of its parts, allowing emergent behavior related to correlated spatial and temporal structures [12,13,14]. As seen, this integration provides the needed structure for more complex organisms [15,16,17,18,19].

The integrated information theory (IIT) provides the mathematical framework using information-theoretic principles in which it is attempted to measure the degree of interconnection of the parts of a system in terms of information exchange [20,21,22,23], where the information typically arises from causal interactions. Originally IIT is related to the conscious experience and its different mathematical descriptions have been adopted also as quantitative measure for complex dynamics [14,21,24,25,26,27].

To the best of our knowledge there is a lack of literature following this approach in genetic systems. Existing studies using information theory include the application of methods such as entropy and mutual information for network reconstruction [28,29,30]. These measures are also applied for research focused on the architecture and function of gene networks, where the network functionality is computed through the channel capacity and rate of signal transmission between regulatory signals and effector proteins [31]. In signaling systems this includes the determination of the amount of information transfer and reliability of signal transduction [32]. Some other applications include complex identification and resilience of network in protein-protein interactions [33] as well as reverse engineering and structure-dynamics identification in metabolic networks [34].

The proposed study based on IIT measures is of interest as it provides a practical application of sophisticated methods to quantify integration of information, which is fundamental for cell communication. Beyond neural systems, signal transduction holds mechanisms that integrate stimuli to achieve functionality in cells. Using computational modelling based on gene regulation we could account for mechanisms of intracellular and extracellular organization including multistability, stochastic gene expression, oscillations, pattern formation, among others. This is also of interest to understand emergent phenomena and optimization in the process of information exchange, and it could be also extended to design principles to more standard synthetic construction processes.

We address this question by studying an ensemble of existing synthetic repressilators. The repressilator network is an example of an engineered genetic system with predictive properties through modeling of its transcriptional regulation [35]. As a single cell, it is inferred that the protein concentrations oscillate over time. This has been observed experimentally with spontaneous oscillations with a period less than the division cycle. However, in realistic living systems cells do not act in isolation, but through cooperative interactions using signaling networks [36]. Thus, the intercellular communication enables the system to exhibit a rich repertoire of dynamical solutions reflecting phenotypic states, which may account for adaptation and differentiation [5,37]. In previous research, to understand cell-cell interaction the repressilator was coupled with other genetic oscillators through signaling molecules (acylated homoserine lactone) largely dependent on the population density. It was found that increasing the coupling among cells with different periods allowed them to reach phase-locking synchronization. The same system could be rewired using a different signaling pathway [38] in such a way that coupling exhibits for increasing coupling phase-repulsive ending with oscillation death. At the same time, multistable regimes appear with the possibility of different cluster distributions and emergence of chaos [38,39,40,41]. All these dynamical regimes could be interpreted as diversification of phenotypic states. This implies the coexistence of different communities in which the oscillators exhibit similar behavior, whose grouping ability is influenced by the degree of chaos.

In this paper, we compute integrated information (II) using the repressilator circuit coupled via quorum sensing. We use three different measures of II, namely, decoder-based integrated information [13], stochastic interaction [12,14] and whole-minus-sum integrated information [42,43]. This is particularly insightful as there are many candidate measures and still it is missing a consensus about their applicability and relationship in dynamical systems. Thus, we show the different levels of complexity that appear in terms of the signaling architecture and cell coupling. First, in the phase-attractive coupling scheme we calculate II in the transition to synchronization as a function of the cell density in a noisy population of genetic oscillators. It is found that the considered measures of II can detect order-disorder transitions exhibiting maximal susceptibility at self-organized criticality. In the case of phase-repulsive coupling, II is calculated for cells that exhibit different oscillatory clusters that change its distribution over time in terms of the degree of chaos. We explore the properties of standard measures of II and compare their behavior across these dynamical regimes. Our results give insights to understanding their applicability and to which extent each one reflects some degree of dynamical complexity.

### 1.1. Coupled Repressilators

**Figure 1:**
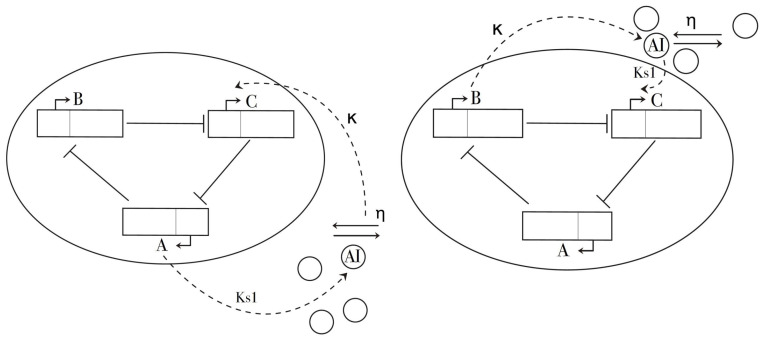
Diagram of the repressilator circuit including the quorum sensing mechanism. **Left**: the phase-attractive coupling. **Right**: the phase-repulsive coupling.

Introduced from the seminal work [44], the repressilator is an artificial genetic network formed of three genes interacting through a negative feedback loop. This is, the activity of each gene is repressed in a cyclic manner: gene tetR encodes the TetR protein, which in turn, represses the transcription of protein CI produced from gene cl. Finally, CI binds to laCI to inhibit gene LacI. As mentioned previously, as a single cell, the repressilator network can produce oscillations, but here we are interested in including the coupling to understand the dynamics of multicellular systems. This can be modelled using a quorum sensing module using bacterium vibrio fischeri composed of two proteins LuxI and LuxR as suggested initially in [17,35]. Protein LuxI synthesizes an autoinducer (AI) that encodes the coupling among the neighboring cells. AI molecules diffuse throughout the extracellular space, and forms the complex LuxR-AI that binds to lacI, which in turn inhibits the expression of gene tetR. Under this general mechanism, different architectures are possible in terms of the gene from the repressilator circuit that regulates the concentration of LuxI. This enables the multicellular system to exhibit different dynamics that would not be observed in an isolated cell. In this work we concentrate on two coupling schemes, in particular, the phase-attractive and phase-repulsive coupling. Both descriptions are illustrated in Figure 1.

### 1.2. Phase-Attractive Coupled Repressilators

Consider a multicellular system in which each cell contains a repressilator network. Following the description from above, the repressilator is coupled through a quorum sensing mechanism in which AI molecules diffuse from the cellular membrane to other cells inducing the activity of a second plasmid lacI. In this way, the mRNA dynamics can be described by the following set of equations

(1) $$\begin{array}{cc} \frac{da_{i}}{dt} & =-a_{i}+\frac{\alpha }{1+C_{i}^{n}}\\ \frac{db_{i}}{dt} & =-b_{i}+\frac{\alpha }{1+A_{i}^{n}}\\ \frac{dc_{i}}{dt} & =-c_{i}+\frac{\alpha }{1+B_{i}^{n}}+\frac{\kappa S_{i}}{1+S_{i}} \end{array}$$

where $a_{i}$, $b_{i}$ and $c_{i}$ denote the mRNA concentrations in cell i=1,...,N for genes tetR, cI, and laCI, respectively. Each mRNA transcripts proteins with concentration levels denoted by $A_{i}$, $B_{i}$ and $C_{i}$. The concentration of the AI in cell *i* is denoted by $S_{i}$. Here mRNA and protein concentrations have been rescaled by their degradation rate (assumed equal for the three genes). The cooperativity number is denoted by the Hill coefficient *n*, and the maximum transcription rate is denoted by α. Finally, κ describes the maximum transcription amount of lacI by saturated concentration of AI.

The protein concentration in cell *i* evolves according to

(2) $$\begin{array}{c} \frac{dA_{i}}{dt}=\beta_{a}\left(a_{i}-A_{i}\right)\\ \frac{dB_{i}}{dt}=\beta_{b}\left(b_{i}-B_{i}\right)\\ \frac{dC_{i}}{dt}=\beta_{c}\left(c_{i}-C_{i}\right) \end{array}$$

where $\beta_{a,b,c}$ denotes the ratio between mRNA and protein lifetimes for each respective gene, tetR, cl, and lacl, respectively.

Observe that the architecture in the multicellular system is provided by the coupling scheme. To get phase synchronization we assume that the production of LuxR is governed by the expression of lacl. Thus, there is a reinforcement in the concentration of LacI as we increase the production of AI molecules.

Using this condition, the evolution for the AI is expressed by

(3) $$\frac{dS_{i}}{dt}=k_{s0}S_{i}+k_{s1}A_{i}-\eta \left(S_{i}-S_{e}\right)$$

here we assume a first order degradation, synthesis and intercellular diffusion encoded by the parameters, $k_{s0}$, $k_{s1}$ and η, respectively. The diffusion rate of AI through the cell membrane η can be expressed as $\eta =\delta /V_{c}$, where $V_{c}$ is the cell volume and δ encodes the membrane permeability and surface area. The amount $S_{e}$ represents the extracellular concentration of AI, which in the quasi-state regime the mean field approximation can be written as $S_{e}=Q\bar{S}$, where $\bar{S}=\sum_{j=1}^{N}S_{j}/N$ denotes the average AI concentration over all cells in the system. The coupling *Q* is linearly dependent of the cell density $\delta N/V_{ext}$ in the system through the expression $Q=\delta N/V_{ext}/\left(k_{se}+\delta N/V_{ext}\right)$, where *N* is the number of cells, $V_{ext}$ the extracellular volume and $k_{se}$ the AI degradation rate.

**Figure 2:**
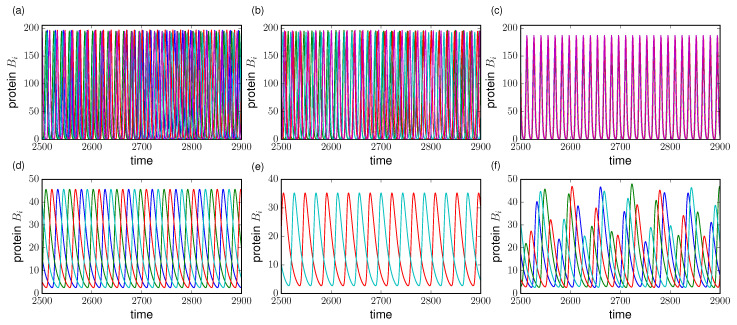
Time series of protein levels $B_{i}$ for different coupling *Q*. (**a**–**c**) phase-attractive coupling with N=100 and κ=20. Cell variability is in terms of lifetime ratios $\beta_{a,b,c}$∼N(1,0.003); (**d**–**f**) phase-repulsive coupling with N=4 cells, κ=25, $\beta_{a}=0.85$, $\beta_{b,c}=0.1$; (**a**,**d**) Q=0.4, (**b**,**e**) Q=0.57 and (**c**,**f**) 0.8. Other parameters are α=216, n=2.6, η=2.0, $k_{s0}=1$ and $k_{s1}=0.01$. (**a**–**c**) describe the transition to synchronization by increasing the coupling *Q*, (**d**–**f**) show the transition from stable clusters of regular oscillations to a chaotic behavior. Each color line represents the protein dynamics of each cell in the network.

Figure 2a–c shows the transition to the phase-locking state as we increase the cell density through the coupling parameter *Q*.

### 1.3. Phase-Repulsive Coupled Repressilators

If we rewire the quorum sensing pathway by setting the gene luxI repressed by the activity of protein TetR we obtain phase-repulsive coupling. This is a consequence of the competition between the induced negative feedback created by tetR and lacI and the repressilator circuit. In addition, the repression of CI favors the production of luxI. Then, by including these observations (3) can be modified as follows

(4) $$\begin{array}{c} \frac{dS_{i}}{dt}=k_{s0}S_{i}+k_{s1}B_{i}-\eta \left(S_{i}-S_{e}\right) \end{array}$$

In systems where N≥2 it has been identified several attractors that include phase-shifted oscillations, clustering and chaos (Figure 2d–f, respectively) for increasing cell density. Thus, by defining the architecture for cell signaling there are implications in the emergence of cooperative behavior with different phase relations. If we relate this behavior with actual cellular states, understanding the different solutions is of crucial importance to get an insight into cell differentiation between different fates, its variability and robustness. In this work, we follow the discussion provided by [39,40] by tuning the cell density through the coupling *Q*. In particular, we focus on a dynamical regime that encompasses clustering, i.e., synchronized oscillation in subgroups, followed by chaos between two torus bifurcations.

### 1.4. Information-Theoretic Measures

We explore the spatio-temporal interactions of genetic oscillators that communicate through quorum sensing. The concentrations of mRNAs and proteins oscillate with a specified period and amplitude exhibiting different configurations in terms of the signaling architecture and biological parameters. In this work we choose the protein *B* to encode the dynamic state of each cell (due to the model symmetry this has minor effects in the results). In turn, oscillations from individual cells are binarized using as a threshold the mean of its instantaneous amplitude from the Hilbert transform. In other words, the absolute value of the analytical signal $L_{H}\left[B\right]$ defined as

(5) $$L_{H}\left[B\right]=B\left(t\right)+iH_{B}\left(t\right)=L_{B}\left(t\right)e^{i\theta (t)}$$

where

(6) $$H_{B}\left(t\right)=\frac{1}{\pi }P.V.\int \frac{B(\tau )}{t-\tau }d\tau$$

is the Hilbert transform (P.V. denotes the Cauchy principal value). Thus, we can write

(7) $$X\left(t\right)=\left\{\begin{array}{cc} 1, & B\left(t\right)\geq \langle L_{B}\left(t\right)\rangle \\ 0, & \mathrm{otherwise}\; \end{array}\right.$$

where X(t) denotes the binarized state of B(t).

This is useful to the general picture of gene activation in regulatory networks, in which every gene switches between a high or low state in terms of the interplay of activation and inhibition of the chemical signals of the other genes in the network. Therefore, this procedure naturally can be applied to other kind of gene dynamics in which a transition between states is considered. This approach for binarization has been followed for recent work in neuroscience [45]. In this paper, we integrate numerically the set of equations that defines the time evolution of the system and then by binarizing each channel from the solution we obtain a dataset where at each time step the state consists of a bit array of length equal to the number of channels.

Formally, let *X* to be a multidimensional stationary time series composed of binary information channels $X_{i}$, where i=1,...,N with *M* observations (time steps) each. Then each state *x* is a bit array of *N* bits. Consider *Y*, the lagged signal of process by τ units of time. The current state of *X* is denoted by $X_{t}$ and *Y* by $Y_{t}=X_{t+\tau }$. The time-delayed mutual information (TDMI) is defined as

(8) I(XY)=H(X)+H(Y)−H(XY)

where XY denotes a joint event, expressed as the concatenation of two-bit arrays *X* and *Y*. The Shannon entropy $H\left(X\right)=-\sum_{x}p\left(x\right)\log p\left(x\right)$ quantifies the average number of bits needed to encode a state of the system [46]. Observe that *H* is time-independent because of the stationarity assumption. TDMI provides a measure about causal correlations of a many-body system. This is given in terms of the reduction of uncertainty of state at t+τ given the evolution up to *t* steps, i.e., it provides the uncertainty reduction about the future state by knowing the present states (or vice versa). In this way, TDMI quantifies the predictability in the evolution of the entire system. This complexity alone has proven to be useful in as a flexible tool, from identifying nonlinear correlations and phase transitions to network inference in genetic networks [30]. TDMI implies that we decompose the system *X* in two signals, which represent the evolution of same process, but are separated by a time lag. In this way, spatio-temporal interactions X→Y are lost, and we measure the degree of causal correlations between them. We are also interested in considering the interaction between the components of the system. Then we now partition the system into subsystems containing a defined number of channels in each one, and measure how tightly interconnected the subsystems are in terms of information exchange. In our case using binary time series the full set of *N* bits can be now divided into non-overlapping non-empty subsets of bits. Such a splitting is commonly a bipartition Π=AB such that $X=X_{A}X_{B}$, due to computational tractability. Other partition scheme is the atomic partition, in which every subsystem contains one channel only. This latter one provides an upper bound for the measure of integration in a system, because we neglect the interactions of every $X_{i}$, for i=1,...,N. Thus, this scheme provides the maximum information loss.

IIT has proposed different versions to measure integration of information, in which the fundamental idea is to quantify the loss of information by assuming the whole system partitioned in its components [13,20,21,22]. Different complexities have been suggested as potential measures of integrated information expressed as the distance between the probability distribution of the actual system and the product of probability distributions associated with its parts, provided some theoretical constraints [14]. Several measures have been derived following this approach although the empirical calculation is not plausible in some cases due to theoretical conditions and computational demands. In this paper, we examine three different measures which allow practical computations from experimental data using empirical distribution probabilities.

The first measure we use is the whole-minus-sum integrated information introduced by Barrett and Seth $\Phi_{WMS}$ [42,47], defined as

(9) $$\Phi_{WMS}\left(XY\right):=\Phi_{WMS}\left(XY;\mathrm{MIB}\right)$$

where $\Phi_{WMS}$ denotes the effective information

(10) $$\Phi_{WMS}\left(XY;\mathrm{AB}\right):=I\left(XY\right)-\left(I\left(X_{A}Y_{A}\right)+I\left(X_{B}Y_{B}\right)\right)$$

MIB∈AB is the bipartition with the minimum information loss quantified by the mutual information between groups of channels.

This measure can be interpreted as the amount of integration of the components of a system through the creation of information when the system acts as a whole as compared with the information generated by its parts. However, it has been criticized since it does not satisfy the positivity requirement of IIT which makes it difficult for interpretation. Still, we find it plausible for discussion with more sophisticated approaches since it determines, from an information-theoretical point of view, the degree of synergetic or redundant influences of the whole system in its evolution. Thus, giving a practical insight to the global integration of causal interactions.

Another measure is the decoder-based integrated information $\Phi^{*}$ proposed in [13]. As in (10), this quantity aims to measure global integrations through the difference of mutual information between the actual system and a partitioned one, where the parts are assumed independent, and satisfies the theoretical postulates of IIT. Thus, $\Phi^{*}$ quantifies the extent of information loss when connections in the system are neglected through a mismatched probability distribution. This can be written as follows,

(11) $$\begin{array}{cc} \Phi^{*}\left(XY;\mathrm{MIB}\right) & :=I\left(XY\right)-I^{*}\left(XY;\mathrm{MIB}\right)\\ I^{*}\left(XY;\mathrm{MIB}\right) & =\max_{\beta }\tilde{I}\left(XY;\beta \right) \end{array}$$

where $\tilde{I}$ denotes the mismatched decoding through the expression

(12) $$\begin{array}{c} \tilde{I}\left(XY;\beta ,\mathrm{MIB}\right)=-\sum_{X}p\left(X\right)\log\sum_{Y}p\left(Y\right)q{\left(X|Y\right)}^{\beta }\;\\ +\sum_{X,Y}p\left(XY\right)\log q{\left(X|Y\right)}^{\beta } \end{array}$$

where β is a parameter obtained from a gradient descent approach, and q(X|Y) denotes the mismatched decoding probability distribution defined for bipartitions AB as

(13) $$q\left(X|Y\right)=p\left(X_{A}|Y_{A}\right)p\left(X_{B}|Y_{B}\right)$$

Finally, the stochastic interaction $\tilde{\Phi }$ introduced in [12] is defined as

(14) $$\tilde{\Phi }\left(XY;\mathrm{AB}\right)=H\left(Y_{A}|X_{A}\right)+H\left(Y_{B}|X_{B}\right)-H\left(Y|X\right)$$

It quantifies the global stochastic interaction in the system dynamics measured through the total uncertainty reduction of each of its parts during a transition to future states, where it is assumed that each part at each time step has knowledge of its own state and its complementary. It can also be interpreted as the extent of how tightly all parts of a system are in its dynamics in terms of information exchange more than the sum of its parts.

It can be shown that (14) can be written as

(15) $$\tilde{\Phi }\left(XY;\mathrm{AB}\right)=\Phi_{WMS}\left(XY;\mathrm{AB}\right)+I\left(Y_{A}Y_{B}\right)$$

In this way, the stochastic interaction encodes the spatial correlation $I(Y_{A}Y_{B})$ measured through the mutual information between halves of the system and the net synergy.

For this work, we use the protein concentration of a chosen gene of the repressilator circuit from each cell to encode the global dynamics in the network. Thus, for each time step, the global state will be defined as a binary segment of length *N*, where each digit represents the binarized state from an individual cell using the binarization scheme previously explained (7). Each state has been assigned with a probability of occurrence, and $2^{N}$ is the maximum number of possible states for time step. Finally, for the integrated information measures were computed using the ’Practical PHI toolbox’ and we apply the Queyranne algorithm [13,14,48,49] for the MIB search.

### 1.5. Synchronization Properties

The coupled repressilators with quorum sensing exhibit several oscillator configurations: from in-phase behavior, splay-states, chaos, and clustering. We consider the generalized order parameters [50,51,52]

(16) $$R_{k}\left(t\right)={\langle e^{ik\theta_{j}\left(t\right)}\rangle }_{j},j=1,...,N,k\in N$$

where $\theta_{j}\left(t\right)$ is obtained from (5). Thus, it is possible to quantify the degree of k-cluster synchronization and asymmetries. In addition, the fluctuations in the order parameter can be computed through its variance

(17) $$\sigma^{2}\left(R_{k}\right)={\mathrm{var}}_{t}R_{k}\left(t\right)$$

## 2. Results

We show the plots of the measures of integrated information described above from which we contrast their differences for the specific genetic repressilator architectures. For each coupling scheme we show the qualitative trends for each measure in terms of the cell density encoded in the coupling and system size and compare the results with the underlying dynamics assessed by other standard approaches. The analysis will be split according to the different types of coupling.

In each case, the time series are generated from Equations (1) and (2), where $S_{e}$ is determined from (3) (phase-attractive coupling) or (4) (phase-repulsive coupling). A fourth order Runge-Kutta method with randomized initial conditions is used for integration. We select a step size of 0.05 and $1.5\times 10^{6}$ steps from which we remove d $5\times 10^{5}$ time steps to avoid transient effects. Probabilities are computed from the frequency counted empirically from where we get all the information-theoretic measures in bits.

The results presented in the following sections consider the bipartition scheme unless it is stated otherwise. Due to explanatory purposes when we discuss the integrated information as a function of the system size we will use the atomic partition. The reason comes from the intractability of computing in a reasonable time any candidate measure of integrated information for larger values of *N*. However, from the simulations in this paper we find that the qualitative trends are the same for intermediate sizes *N*, being just slightly higher than the bipartition scheme. This is because the atomic partition allows to maximize the informational loss as the system is split completely [13,53]. In these terms we can regard this measurement as an upper bound for the integrated information using any other partition scheme. Moreover, cell populations appear as interacting elements in large scale. Thus, it is worth understanding the influence of the size in the balance of segregation and integration of the system dynamics, which can be computed through the assumption of a split system in all its parts.

### 2.1. Phase Synchronization

**Figure 3:**
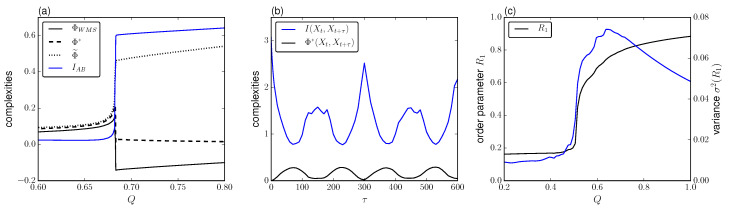
(**a**) Mutual information $I_{AB}$ and integrated information measures $\Phi_{WMS}$, $\Phi^{*}$ and $\tilde{\Phi }$ for increasing coupling *Q*. (**b**) Mutual information $I(X_{t},X_{t+\tau })$ and $\Phi^{*}$ for increasing τ and Q=0.5. (**c**) Order parameter $R_{1}$ and its variance for increasing *Q*. Other parameters are the same as in Figure 2 for the phase-attractive coupling.

In realistic conditions the population of repressilators exhibits important diversity in its oscillatory behavior as a consequence of cell diversity. Following [35], the lifetime ratio β plays a key role in defining the period length. Thus, we encode this diversity within the community by assuming β non-uniformly distributed. In particular, we choose a normal distribution with mean 1.0 and standard deviation Δβ. What we observe is a set of oscillations with a broad distribution of uncoupled phases. For increasing coupling there is a transition to phase synchronization, such that the larger is the variation in β the larger *Q* we need to reach the phase-locking state. In Figure 3c we show as complexity measure the variance of the order parameter $R_{1}$ defined in (17) averaged over $10^{3}$ oscillators for different *Q* and $\Delta \beta^{2}=0.005$. The system has negligible fluctuations for small coupling which are maintained constant as intercell effects are not strong enough. As cell density increases, there is a partial phase-locking which increases suddenly to complete synchrony. At the same time, the fluctuations in $R_{1}$ span abruptly from zero to its maximum value around the transition regime at Q≈0.68. This range of transition agrees with the García-Ojalvo’s order parameter reported with the original model.

#### 2.1.1. Integration and Synchronization

Next, we take N=4 cells to exemplify to which extent the synchronization process is captured by the measures of integrated information. In Figure 3a we see the measures drawn from Equations (9)–(15) using τ=300 time steps, corresponding to approximately the mean period of oscillation of the cell population. As observed, the measures $\Phi_{WMS}$ and $\Phi^{*}$ monotonically increase from zero reaching its peak as the transition point is approached and drop sharply with similar behavior after the critical point. Then, the whole-minus-sum measure $\Phi_{WMS}$ reaches negative values while the decoder-based integration drops to zero. The stochastic interaction $\tilde{\Phi }$ follows the same trend as the other Φ measures before the transition regime but increases as the system synchronizes.

Thus, both $\Phi_{WMS}$ and $\Phi^{*}$ indicate that at the transition point to phase synchronization, the system is responsive to its own states more than the aggregation of the individual responses. After reaching synchronization there is high integration and segregation is negligible. As a consequence, these measures show a drop to lower values. This is not the case for $\tilde{\Phi }$, which seems to increase as the system integrates more. It is worth understanding the influence of the spatial segregation and integration in the dynamics, to relate it with our previous results. We quantify the mean mutual information between all possible halves of the system for increasing coupling denoted as $I_{AB}=I\left(X_{A}X_{B}\right)$, Equation (8) (Figure 3a). As expected, for small values of coupling, there is small correlation which is enhanced during the phase transition with a step-like trend. Next, it reaches a plateau in the strong coupling regime. Thus, we see that $\tilde{\Phi }$ is dominated by the behavior of the spatial correlation in the process $X_{t}$, $\Phi_{WMS}$ interprets the high correlation of the synchronized system as redundancy, and $\Phi^{*}$ vanishes due to high synchronization between cells.

The trends described above depend on the lag τ under consideration. In particular, we observe two main cases: for τ where the TDMI is maximum, the measures $\Phi_{WMS}$ and $\Phi^{*}$ are maximized at criticality, while for τ where the TDMI is minimum, the is a general tendency of $\Phi_{WMS}$ and $\Phi^{*}$ to decrease towards the super-critical regime. Meanwhile, $\tilde{\Phi }$ keeps the same behavior already described for any lag.

**Figure 4:**
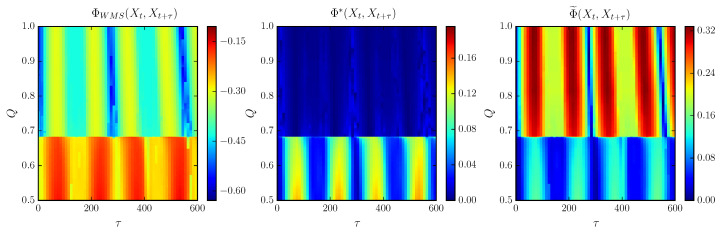
Measures of integrated information for increasing *Q* and τ. Lifetime ratio β∼N(1,0.005). Other parameters are the same as in Figure 2 for the phase-attractive coupling.

Figure 4 shows the transition depicted by $\Phi_{WMS}$, $\Phi^{*}$ and $\tilde{\Phi }$ in terms of *Q* and τ for $\Delta \beta^{2}=0.005$. When considering the relationship with the mutual information $I(X_{t},X_{t+\tau })$ we observe that the integrated information measures are maximized when the TDMI is minimized, i.e., when $X_{t}$ and $X_{t+\tau }$ have the least redundancy. This is the situation where $X_{t}$ and $X_{t+\tau }$ are out-of-phase. In a similar way, the integrated information measures are minimized when $X_{t}$ and $X_{t+\tau }$ are in-phase. This holds true for all values of *Q* for $\Phi_{WMS}$ and $\tilde{\Phi }$.

In the case of $\Phi^{*}$, the aforementioned feature is satisfied before synchronization, but after the phase transition it vanishes for all τ. Thus, from the perspective of $\Phi_{WMS}$ and $\Phi^{*}$ the whole system has more information about its current state given its past than its individual components when $X_{t}$ and $X_{t+\tau }$ are sufficiently independent but still preserving some degree of correlation.

#### 2.1.2. Integration and Scaling Behavior

**Figure 5:**
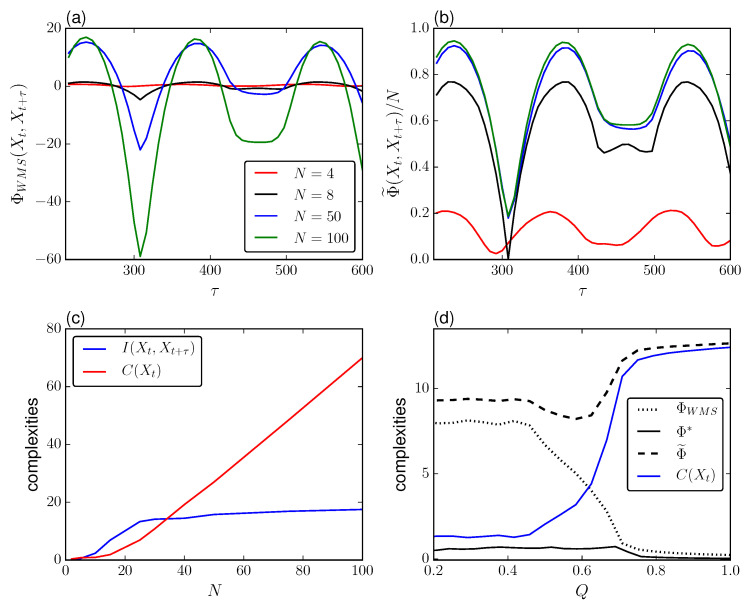
(**a**) Whole-minus-sum integration $\Phi_{WMS}$ and (**b**) stochastic interaction $\tilde{\Phi }$ for increasing τ for different system sizes *N*. (**c**) Mutual information $I(X_{t},X_{t+\tau })$ and total correlation $C(X_{t})$ for increasing size *N*. (**d**) Integration measures $\Phi_{WMS}$, $\Phi^{*}$ and $\tilde{\Phi }$ for increasing coupling *Q*. For comparison we include $C(X_{t})$. For (**a**–**c**) we use Q=0.5 and (**c**,**d**) we use τ=60 units with β∼N(1,0.005). Other parameters are the same as in Figure 2 for the phase-attractive coupling.

In general, the qualitative trends shown in Figure 4 hold regardless of the system size. However, it is worth discussing the influence of the TDMI and spatial correlation in the measures of integrated information. In Figure 5a,b we show the whole-minus-sum integration and the normalized stochastic interaction across different sizes *N* and lags τ. As discussed previously, it is observed that regardless of the system size the integration measures are symmetric with respect to the TDMI. In particular, when the TMDI is minimized, $\Phi_{WMS}$ grows with a concave downward trend for increasing *N* while for lags τ in which the TDMI is maximized, $\Phi_{WMS}$ becomes increasingly negative. In contrast, $\tilde{\Phi }$ keeps a linear growth independent of τ for large *N* with fixed coupling. This means that if $X_{t+\tau }$ encodes the most possible information of $X_{t}$, the integrated information will be maximized, reaching a plateau for increasing system size. Meanwhile, if $X_{t}$ and $X_{t+\tau }$ are highly correlated, the components of the system will hold more information about their own state than the system.

Next, we study how the different measures change for increasing *Q* using the first lag that minimizes the TDMI, τ=60 to maximize the amount of influence between $X_{t}$ and $X_{t+\tau }$. Figure 5d shows different complexities, including the integrated information measures and the total correlation defined as $C\left(X\right)=\sum_{i=1}^{N}H\left(X_{i}\right)-H\left(X\right)$ for an intermediate size N=15 (observe that C(X) replaces $I(X_{A}X_{B})$ in Equation (14) when we consider the atomic partition). As it is shown, for coupling *Q* before the transition to synchronization $\Phi_{WMS}$ and $\Phi^{*}$ remain steady followed by a drop to a low value of integrated information just right after the cutoff point for synchronization. Thus, at the transition point, both measures are maximally sensitive and decrease in the super-critical regime where the system loses segregation.

Interestingly, the decoder-based integration has a negligible integrated information when compared with the whole-minus-sum integration despite showing a similar trend. The dependency between them is not entirely clear, but one can infer that the mismatched decoding is assuming that as the system grows, the patterns in the sub-critical region are noise driven due to the uncoupling between cells (high segregation) followed by a transition to a high correlated regime (high integration). This is out of the scope of this paper and further investigation is needed.

Meanwhile, $\tilde{\Phi }$ remains steady for *Q* spanning from zero to just before criticality, then it dips around the transition point, and stabilizes and remains constant at a higher value of integrated information with respect to the unsynchronized state. In comparison with $\Phi_{WMS}$ and $\Phi^{*}$, this measure is dominated by *C* for large *N*, which scales linearly, Figure 5c. On the other hand, $\tilde{\Phi }$ dips because of the contribution from $\Phi_{WMS}$ is reduced while the total correlation *C* increases due to the phase synchronization.

From a biological perspective these results suggest that the coupling at the transition point to synchronization would be favored to keep a balance between integration and differentiation of the regulatory dynamics in a coherent way. High integration leads the system to exhibit split dynamics while some degree of segregation provides evolutionary advantage to the whole system to act to internal and external demands.

### 2.2. Phase-Repulsive Coupling

#### 2.2.1. Irregular Self-Oscillations

**Figure 6:**
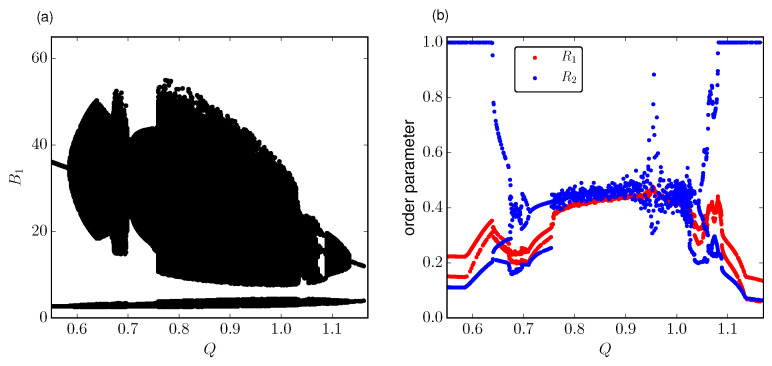
(**a**) Numerical bifurcation plot for the full amplitude oscillations and (**b**) order parameter $R_{1,2}$ for different values of coupling *Q*.

In Figure 6a we show the numerical simulations of the bifurcation plot for N=4 cells for the model (Section 1.3). It is observed regular oscillations which are completely out-of-phase up to around Q=0.49 followed by the stable formation of subgroups of synchronized cells (clustering). As we reach the first torus bifurcation $TR_{1}$ around $Q^{*}=0.58$ there is emergence of chaos reflected in oscillations of amplitude with increasing irregularity [40]. This behavior is maintained just before the second bifurcation point $TR_{2}$ around Q=1.13 in which regular oscillations are recovered. This behavior is observed across different number of cells for N≥4 for this analysis.

As a proof of concept, we characterize the cluster distributions through the 1,2-cluster synchronization (16) and relate it with the measures of integrated information. From previous results about clustering of coupled repressilators [40], clusters are defined in terms of the absolute difference of the concentration levels of the signaling molecule $S_{i}\left(t\right)$. Here, the cluster ordering is measured from $R_{1,2}$ using the phase of protein oscillations $B_{i}\left(t\right)$. However, the main feature about the cluster dynamics is equivalent to that described by the [40]. This is the enhanced switching from one cluster distribution to another for increasing degree of chaos, as we describe below.

Figure 6b shows $R_{1,2}$ order parameters for increasing *Q*. Each data point indicates the degree of 1,2-cluster synchronization averaged in time. As discussed previously in [41], clusters can be either stable and therefore not changing its composition along the time, or transient, which implies the formation of an initial cluster that is later decomposed after some time steps in another arrangement and so forth. The full history of formation and decomposition of clusters is uniquely determined by the initial state. In addition, the lifetime of one cluster before switching to another arrangement will depend on its degree of stability. Consequently, we get multiple branches for increasing *Q* with each branch corresponding to a basin of attraction. Hence, provided some initial conditions the oscillators settle down to different cluster distributions with a defined degree of order.

Now, following $R_{2}$ (Figure 6b) we observe that one group displays fully synchronous behavior, corresponding to a 2:2 cluster as N=4, while other keeps its phase unsynchronized. The lower branch slowly rises at $Q^{*}$ followed by the appearance of two new groups at Q=0.64. Meanwhile, there is a sudden decrease of the upper branch. Later, these clusters are broken in multiple branches between Q=0.67 and Q=0.75. Up to this point we see that the stability of the arrangement between oscillators is continuously reduced for increasing *Q* as a response of the enhanced formation and decomposition of clusters in some time span, so the temporal average synchronization is reduced for phase-locked 2:2 clusters. This trend is maximized at Q=0.75 since right after this value we observe a sequence of points which defines a very disperse and irregular branch just before Q=0.95. This region corresponds to the fully developed chaos, in which there is a continuous formation and decompositions between cluster groups for an initial distribution of oscillators. As chaos increases the lifetime of such clusters is severely shortened [40] followed by the switch to new distribution throughout the time. This grouping ability losses stability to become more sensitive to initial conditions implying an irregular shape. Around Q=0.95 there is a peak to complete 2-cluster phase-locking, which suggests a narrow window in which chaos reverts to its periodic motion as a 2:2 cluster. Finally, there appear again multiple branches leading to the transition towards the initial regular oscillations. On the other hand, order parameter $R_{1}$ provides the degree of nonuniform distribution of oscillators in different clusters. As in the case of $R_{2}$, we see the emergence of branches leading to a unique group with high asymmetry as chaos is increased. In contrast, lower values of $R_{1}$ are attained in the stable region outside of both torus bifurcation points reflecting larger symmetry in the cluster oscillations.

Finally, if we run simulations for larger system sizes the cluster distributions clearly increase their allowed arrangements as the system size grows. Consequently, the degree of cluster synchrony and asymmetry can follow a very disperse trend instead of well-defined branches even in the stable region, although we point out that the behavior for strong chaos keeps very similar. This fact is reflected in the measures of integrated information as discussed below.

#### 2.2.2. Chaos and Integration

**Figure 7:**
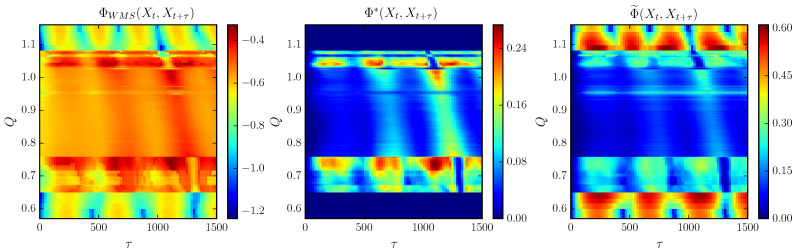
Whole-minus-sum integration $\Phi_{WMS}$, stochastic interaction $\tilde{\Phi }$ and decoder-based integration $\Phi^{*}$ for different values of coupling *Q* and lag τ for N=4 cells. Other parameters are the same as in Figure 2 for the phase-repulsive coupling.

Next, we relate the previous description with the trends followed by the integrated information measures. In Figure 7 we show $\Phi_{WMS}$, $\tilde{\Phi }$ and $\Phi^{*}$ for increasing coupling *Q* and lag τ for N=4.

Compared to Figure 6b one can observe that the integration measures appear to track the broken symmetry due to the formation of new branches for increasing *Q*. First, in the clustering regime $\Phi_{WMS}$ and $\tilde{\Phi }$ hold a periodic trend for increasing τ as a consequence of the regular oscillations of the cluster formations. Within this regime oscillations are highly correlated, so that the stochastic interaction is greater in comparison with the other measures. Meanwhile, $\Phi^{*}$ represents this regime with null integrated information, as the degree of complexity is minimum during synchronization.

**Figure 8:**
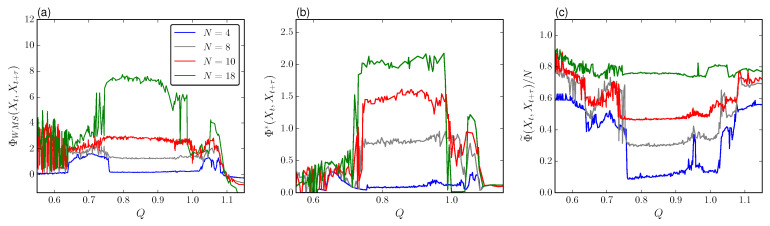
(**a**) whole-minus-sum integration $\Phi_{WMS}$, (**b**) decoder-based integration $\Phi^{*}$ and (**c**) stochastic interaction $\tilde{\Phi }$ for different values of coupling *Q* and different number of cells *N*. We use τ=220 time steps. Other parameters are the same as in Figure 2 for the case of phase-repulsive coupling.

Next, during the onset of chaos, all measures detect the irregular oscillations following a similar trend when we vary τ. However, they differ as the coupling *Q* increases (Figure 8). Both $\Phi_{WMS}$ and $\Phi^{*}$ start with a lower value which is increased non-uniformly along *Q* with the appearance of weak chaos. In turn, there is a drop as the chaotic behavior is enhanced. Meanwhile, $\tilde{\Phi }$ goes from high values of integration due to the stable cluster formation and decreases slightly at $Q^{*}$, followed by a second drop as the spatial interaction collapses to its minimum value.

During the strong chaotic regime all complexity measures decay to zero with an oscillatory trend in the limit τ→∞, since $X_{t}$ and $X_{t+\tau }$ (and the individual system components $X_{i,t}$ and $X_{i,t+\tau }$) are no longer correlated, so the TDMI vanishes. Meanwhile, for the stochastic interaction, the remaining interaction is the spatial correlation encoded in *C*, which seems to be decreased within this regime as shown. Nevertheless, $\tilde{\Phi }$ peaks around Q=0.95 while $\Phi_{WMS}$ becomes more negative and $\Phi^{*}$ vanishes. This suggests a periodic window given that the system becomes highly correlated in the view of $\tilde{\Phi }$ and $\Phi_{WMS}$.

Finally, just before Q=1.1 the maximum amplitudes of the bifurcation plot seem to group in three different regions as observed in Figure 6a, implying an increasing stability ending with the recovery of the periodic behavior after $TR_{2}$. As a result, $\Phi_{WMS}$ and $\Phi^{*}$ increase qualitatively similar during the formation of such groups followed by a drop after the torus bifurcation. In particular, $\Phi_{WMS}$ reaches negative values while $\Phi^{*}$ keeps negligible due to the regular clustering synchronizations. In contrast, $\tilde{\Phi }$ increases progressively reaching high values of integrated information at the torus bifurcation point and stabilizes at a similar value attained just before $TR_{1}$.

#### 2.2.3. Temporal Mixing Increases Integrated Information

As suggested in the past section, the system size plays an important role in defining the degree of dynamical complexity. Again, we make use of the atomic partition to describe the integrated information. Figure 8 shows $\Phi_{WMS}$, $\Phi^{*}$ and $\tilde{\Phi }$ for increasing *Q* at different *N*. In particular, we choose a lag τ=220 time steps, as it keeps close to the first minimum of the TDMI for the range of *Q* considered, so that it reduces as much as possible the redundancy between $X_{t}$ and $X_{t+\tau }$, although both processes are not entirely independent.

Just before $TR_{1}$ we see that for increasing *N* the measure $\Phi_{WMS}$ has a highly disperse arrangement with multiple values of integrated information. This is a result of having several stable cluster distributions as the system grows, each with a certain degree of complexity. Next, after $Q^{*}$, and before Q=0.75, the value $\Phi_{WMS}$ is no longer spread and grows smoothly with *N*. In contrast, for fully developed chaos, $\Phi_{WMS}$ starts with small values increasing with *N* and defines a peak between Q=0.8 and Q=0.95. This is precisely the range in which the chaotic behavior is maximized according to [40].

**Figure 9:**
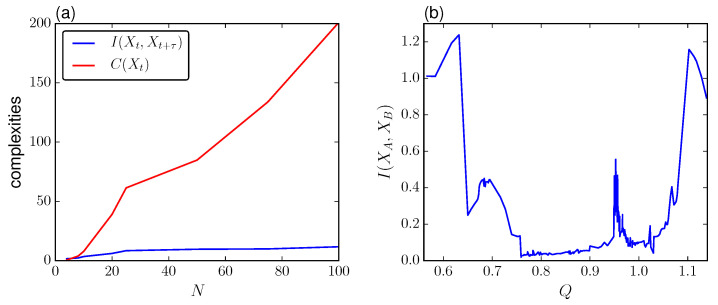
(**a**) Total correlation $C(X_{t})$ and TDMI $I(X_{t},X_{t+\tau })$ for increasing system size *N* and Q=0.7 (weak chaotic regime) (**b**) Mean mutual information between all possible halves of the system $I(X_{A},X_{B})$ for increasing coupling *Q*. We use τ=220 time steps. Other parameters are the same as in Figure 2 for the case of phase-repulsive coupling.

As discussed in the section about phase-attractive coupling, the increasing of $\Phi_{WMS}$ and $\Phi^{*}$ during chaos could be associated with the patterns accessible to the system. The formation and decomposition of clusters favor the accessible states encoded as binary arrays that scale with the system size, but keep upper bounded by the TDMI. Therefore, we also find a saturation of complexity as the system grows as evidenced in Figure 9a. Nevertheless, observe that in all the cases $\Phi_{WMS}$ attributes higher integrated information to the system than $\Phi^{*}$, which means that the information loss due to the mismatch decoding $I^{*}$ from $\Phi^{*}$ is greater. Still, both measures reflect the same properties in the system. This suggests that the multistability provided by the switching of cellular fates increases the balance of segregation and integrations of the causal dynamics of whole system in comparison with the one of its single components leading to the emergence of integrated information. Moreover, this balance has a limiting amount provided by the asymptotic trend of TDMI as the system becomes larger. Next, the whole-minus-sum integration $\Phi_{WMS}$ starts decreasing as the oscillation amplitudes in the bifurcation plot are less spread. In particular, we observe that $\Phi_{WMS}$ drops during the formation of clustered amplitudes in three groups (Figure 6a) followed by a second drop to negative values after $TR_{2}$.

On the other hand, observe that the stochastic interaction $\tilde{\Phi }$ decreases as the number of branches increases. In these conditions the cluster lifetime is shortened implying a decrease of spatial correlations. This effect is quantified by the mutual information between halves $I(X_{A},X_{B})$. As observed the qualitative trend is clearly similar to $\tilde{\Phi }$, so the stochastic information is just reflecting spatial correlation (integration) but not segregation due to the formation of patterns. In addition, as shown in Figure 9a the contribution of the spatial correlation becomes stronger for larger *N* since *C* scales linearly after N=50 cells. Then, from the perspective of the stochastic interaction this rapid switching in the system could avoid synergistic influences on its future states more than the independent oscillators.

## 3. Conclusions

In this work, we assess the degree of integrated information using whole-minus-sum integration, decoder-based integration and stochastic interaction using a model of genetic oscillators coupled by quorum sensing. This work includes two coupling architectures leading to different dynamical regimes representing cellular fates. The phase-attractive coupling allows phase synchronization for increasing cell density, and the phase-repulsive coupling favors cluster formation with increased switching and asymmetry with the degree of chaos.

Our findings suggest that these measures can proxy the transition between dynamical regimes encoded as binary patterns. The whole-minus-sum integration and decoder-based integration are observed to drop in the transition to phase synchronization of cells. In particular, whole-minus-sum integration becomes negative for highly correlated causal interactions between the current and past states. Meanwhile, decoder-based integration vanishes when the system becomes more synchronized. This is because high integration with no segregation implies a low value of integrated information. Thus, these measures have maximal susceptibility near criticality. Also, both measures are increased when there is a balance between the segregation and integration of oscillations due to the transient stability of cellular fates i.e., frequent switching of synchronized clusters, a typical mechanism for decision-making genetic regulatory networks. Both observations suggest that in terms of integration of information, it is evolutionary stable for cells to maintain at criticality prior full synchronization to allow available responses from the cells to intrinsic and extrinsic demands and keeping some degree of integration. In a similar way, the diversity and switching between different attractors not just provide an adaptive advantage to the system but according to our results also maximizes the integration of independent cell dynamics in a coherent way.

Meanwhile, the stochastic interaction, is mainly driven by the spatial correlation and it is not sensitive to the diversity of available states as the other measures considered in this paper. During the transition to synchronization it dips around the critical point due to a combined effect of the decrease of segregation, but it is later enhanced as the system synchronizes favoring full integration. Also, in the chaotic regime, it seems that lower cluster lifetime between switching to new distributions decreases its value, while a larger one favors it. As an overall, for this analysis this measure did not provide better insight than the mutual information between subsystems could.

In all cases, the integrated information measures are symmetric with respect to the TDMI of the whole system. This implies that the balance of segregation and integration of the causal dynamics is favored when the current state of the system shares the less possible information with the past states, but still with some degree of correlation.

Therefore, this suggests that cells, as a system of interacting genetic networks, show enough complexity to exhibit cause-effect power as a whole above and beyond its parts, a required step to develop consciousness according to IIT. However, as we believe, consciousness has not been evolutionarily developed on the genetic level, probably due to the slow communication speed based on diffusion. Further research is required to have a better understanding of the evolutionary mechanisms for higher cognitive processes.
